# Marine Microalgae: Promising Source for New Bioactive Compounds

**DOI:** 10.3390/md16090317

**Published:** 2018-09-06

**Authors:** Caterina R. de Vera, Guillermo Díaz Crespín, Antonio Hernández Daranas, Sofia Montalvão Looga, Katja-Emilia Lillsunde, Päivi Tammela, Merja Perälä, Vesa Hongisto, Johannes Virtanen, Heiko Rischer, Christian D. Muller, Manuel Norte, José J. Fernández, María L. Souto

**Affiliations:** 1 Instituto Universitario de Bio-Orgánica Antonio González (IUBO AG), Centro de Investigaciones Biomédicas de Canarias (CIBICAN), Universidad de La Laguna (ULL), Avenida Astrofísico Francisco Sánchez 2, 38206 Tenerife, Spain; caterina.rdv@gmail.com (C.R.d.V.); gdiazcrespin09@gmail.com (G.D.C.); adaranas@ipna.csic.es (A.H.D.); mnorte@ull.edu.es (M.N.); 2 Departamento de Química Orgánica, Universidad de La Laguna (ULL), Avenida Astrofísico Francisco Sánchez 2, 38206 Tenerife, Spain; 3 Instituto de Productos Naturales y Agrobiología (IPNA), Consejo Superior de Investigaciones Científicas (CSIC), Avenida Astrofísico Francisco Sánchez 2, 38206 Tenerife, Spain; 4 Drug Research Program, Division of Pharmaceutical Biosciences, Faculty of Pharmacy, P.O. Box 56, University of Helsinki, FI-00014 Helsinki, Finland; sofiaimontalvao@gmail.com (S.M.L.); katja-emilia.lillsunde@helsinki.fi (K.-E.L.); paivi.tammela@helsinki.fi (P.T.); 5 VTT Technical Research Centre of Finland Ltd., P.O. Box 1000, FI-02044 Espoo, Finland; merja.perala@auriabiopankki.fi (M.P.); Hongisto@misvik.com (V.H.); johannes.hattara@gmail.com (J.V.); heiko.rischer@vtt.fi (H.R.); 6 Institut Pluridisciplinaire Hubert Curien, UMR 7178 CRNS, Faculté de Pharmacie, Université de Strasbourg, 67401 Illkirch, France; cdmuller@unistra.fr

**Keywords:** bioprospecting, blue biotechnology, marine microalgae, bioactive compound, drug discovery, marine natural products

## Abstract

The study of marine natural products for their bioactive potential has gained strength in recent years. Oceans harbor a vast variety of organisms that offer a biological and chemical diversity with metabolic abilities unrivalled in terrestrial systems, which makes them an attractive target for bioprospecting as an almost untapped resource of biotechnological applications. Among them, there is no doubt that microalgae could become genuine “cell factories” for the biological synthesis of bioactive substances. Thus, in the course of inter-laboratory collaboration sponsored by the European Union (7th FP) into the MAREX Project focused on the discovery of novel bioactive compounds of marine origin for the European industry, a bioprospecting study on 33 microalgae strains was carried out. The strains were cultured at laboratory scale. Two extracts were prepared for each one (biomass and cell free culture medium) and, thus, screened to provide information on the antimicrobial, the anti-proliferative, and the apoptotic potential of the studied extracts. The outcome of this study provides additional scientific data for the selection of *Alexandrium tamarensis WE*, *Gambierdiscus australes*, *Prorocentrum arenarium*, *Prorocentrum hoffmannianum*, and *Prorocentrum reticulatum* (Pr-3) for further investigation and offers support for the continued research of new potential drugs for human therapeutics from cultured microalgae.

## 1. Introduction

We are in a renaissance period for natural product discovery and their potential societal impact as a source of therapeutic drugs is popular [1]. Exponential technology developments in this and other fields are converging towards the provision of innovative pipelines for natural product isolation and identification, their stereo-chemical structure elucidation, biosynthetic origins, and many others that have overcome the difficulties inherent in this area. An exciting field of activity is the development of therapeutics from the marine ecosystem in which the discovery of novel drugs, to date, is small compared to its potential [2,3,4]. Oceans harbor a vast variety of organisms that have developed diverse and unique metabolic abilities resulting in the biosynthesis of a wide number of secondary metabolites with promising biological activities [5,6,7].

Additionally, Blue Biotechnology (or Marine Biotechnology) is an emerging field worldwide based on the use of marine resources either as the source or target of biotechnology applications [8,9]. The outlook for blue biotechnology has changed profoundly over the last decade in large part due to advances in science and technology. This topic encompasses techniques such as bioprocessing, bio-harvesting, bioprospecting, bioremediation, molecular aquaculture, omics approaches, etc., which comprises a horizontal scope and a broad range of subjects [10,11]. A major task of marine biotechnology is to develop an efficient process for the discovery of new and more effective drugs.

Among marine organisms, microalgae play a crucial role in ecosystems as primary producers due to their photosynthetic activity [12]. They are the major producers of biomass and organic compounds in the oceans [6,13] and there is no doubt that they could become genuine “cell factories” for the biological synthesis of bioactive substances with different applications. Despite the fact that, in recent years, there have been an increased number of reports describing novel bioactive compounds produced by microalgae, those represent a relatively untapped source of biologically active compounds [14]. The challenge remains to optimize our capacity to access, identify, and mine them as well as to create the conditions whereby the vast majority of these microorganisms can be cultured to provide a sustainable source of supply. 

As part of our search for marine bioactive compounds, experiments were carried out surveying 33 strains of marine microalgae (dinoflagellates, haptophytas, heterokontophytas, and chlorophyta) with the aim to generate extracts for bioactivity evaluation and face the selection of interesting samples for further investigation. The geographic provenance of these strains was wide (Table S1) and the uni-algal isolates were generously donated by the Oceanographic Center of Vigo (IEO). The strains were cultured at a laboratory scale. Two extracts were prepared for each strain (biomass and cell free culture medium) and, thus, screened to provide information on antimicrobial, anti-proliferative, and apoptotic potential of the studied extracts. These studies were possible within the framework of inter-laboratory collaboration sponsored by the European Union (7th FP) into the MAREX Project (exploring marine resources for bioactive compounds from discovery to sustainable production and industrial application) focused on the discovery of novel bioactive compounds of marine origin for the European industry [15].

## 2. Results and Discussion 

### 2.1. Microalgae Cultures and Extracts Preparation

Clonal cultures of 33 strains that include species of Phyla Dinophyta, Heterokontophyta, Haptophyta, and Chlorophyta were carried out to a medium-scale level to a final volume of 15 L for each strain. The cultures were performed in modified Guillard K medium [16,17] at 22 ± 2 °C and an irradiance of 60 μE/s m^2^ under a 18:6 light:darkness photo cycle. In the last stage, once the stationary growth phase appeared, cultures were incubated statically for 60 days in nutrient depleted conditions. The crude extracts from biomass (species code-C) and cell free culture medium (species code-M) were obtained by maceration/sonication in methanol and by using a solid phase extraction (SPE) on Diaion HP20 resins and methanol, respectively. 

### 2.2. Biological Evaluation 

Extracts from biomass and cell free culture medium obtained for 32 strain batches and from biomass of *Scrippsiella trochoidea* were screened in order to determine their biological properties. Thus, a total of 65 samples were evaluated against selected assays to provide information on antimicrobial, antiproliferative, and apoptotic potential.

#### 2.2.1. Antibacterial, Antifungal, and Antiviral Activity

**Figure 1:**
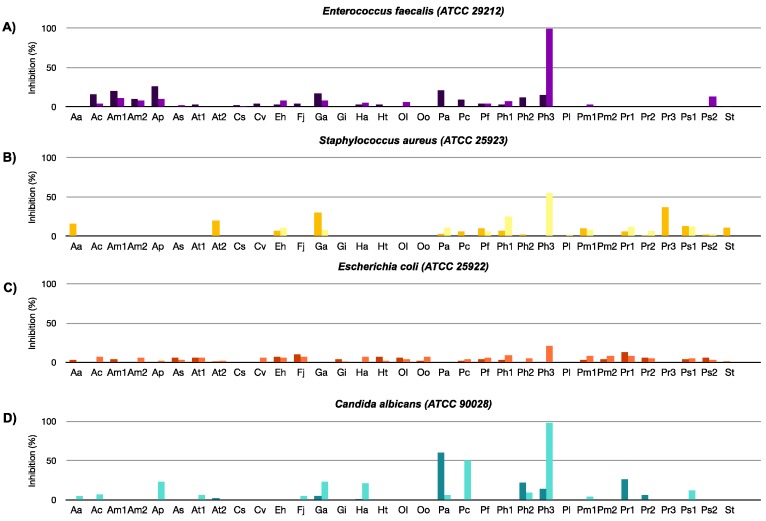
Antimicrobial screening results for microalgae extracts (biomass extracts: dark bars, cell free culture medium extracts: light bars) at 100 µg/mL concentration against: (**A**) *E. faecalis* (ATCC 29212), (**B**) *S. aureus* (ATCC 25923), (**C**) *E. coli* (ATCC 25922), and (**D**) *C. albicans* (ATCC 90028). Ciprofloxacin was used as a reference antibiotic in the antibacterial assays. MIC_90_ (minimum inhibitory concentration) values for *E. faecalis*, *S. aureus*, and *E. coli* were 3, 1.5, and 0.048 μM (1, 0.5 and 0.016 μg/mL), respectively. Amphotericin B was used as a reference in the antifungal assay (MIC_90_ = 0.5 μM (0.5 μg/mL)).

Extracts from biomass and cell free culture medium were evaluated for antimicrobial activity against two Gram-positive bacteria, *Enterococcus faecalis* (ATCC 29212) and *Staphylococcus aureus* (ATCC 25923), one Gram-negative bacterium, *Escherichia coli* (ATCC 25922) and a fungal strain, *Candida albicans (*ATCC 90028). Overall, most of the extracts were inactive against the species studied (Figure 1). However, the extract from *Prorocentrum hoffmannianum 1031* medium (Ph3-M) inhibited the growth of *E. faecalis* and *C. albicans* by 100% and 98%, respectively. This extract showed broad spectrum activity across our assay panel (Table S2) including strong cytotoxicity against several mammalian cell lines. *Prorocentrum* species are known to produce toxins such as okadaic acid [18], which have been demonstrated to possess antifungal activity [19] and could also explain the observed generalized toxicity. It is, however, interesting that some of the other tested *Prorocentrum* species did not have antibacterial or antifungal effects but were active against mammalian cells. This would imply the presence of antimicrobial substances specific for *Prorocentrum hoffmannianum 1031*. Interestingly, some cellular extracts such as *Alexandrium minutum* (Am2-C) and *Heterosigma akashiwo* (Ha-C) were shown instead to significantly enhance the growth of *S. aureus* (Table S2), which may indicate that the extracts included components acting as nutrients for the bacteria.

**Figure 2:**
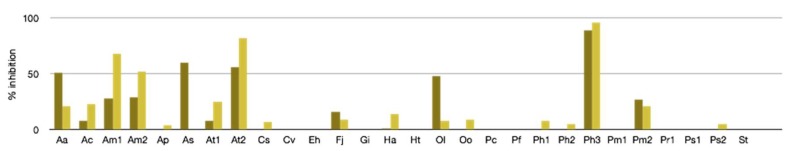
Inhibition of CHIKV replicon (%) of microalgae extracts at 100 µg/mL. Dark bars represent biomass extracts and light bar cell free culture medium extracts.

In addition, the extracts were also studied against the Chikungunya virus (CHIKV) by using a replicon model (Figure 2). Three samples showing potent inhibition activity >80% in the primary screening at 100 µg/mL concentration: *Alexandrium tamarense WE* (At2-M) 82%, *Prorocentrum hoffmannianum 1031* (Ph3-C) 89%, and (Ph3-M) 98%. Since the replicon assay is based on a BHK cell line, cytotoxicity of the active extracts against the host cell was studied to rule out false positives due to host cell cytotoxicity. The cytotoxicities for At2-M, Ph3-C, and Ph3-M were 80%, 92%, and 87%, respectively. Thus, the effects seen in the CHIKV replicon assay were clearly caused by host cell cytotoxicity and not by the inhibition of replication [20]. All three extracts were also found to be active in the antiproliferative assays, which is in accordance with the BHK cytotoxicity results.

#### 2.2.2. Antiproliferative Activity

**Figure 3:**
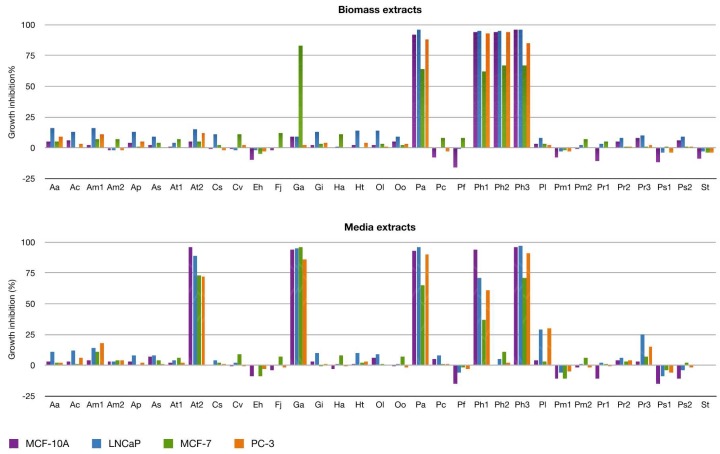
Growth inhibition (%) against cell lines: MCF-10A (breast cells), MCF-7 (breast cancer cells), LNCaP, and PC-3 (prostate cancer cells). All the samples were tested at 50 µg/mL.

**Figure 4:**
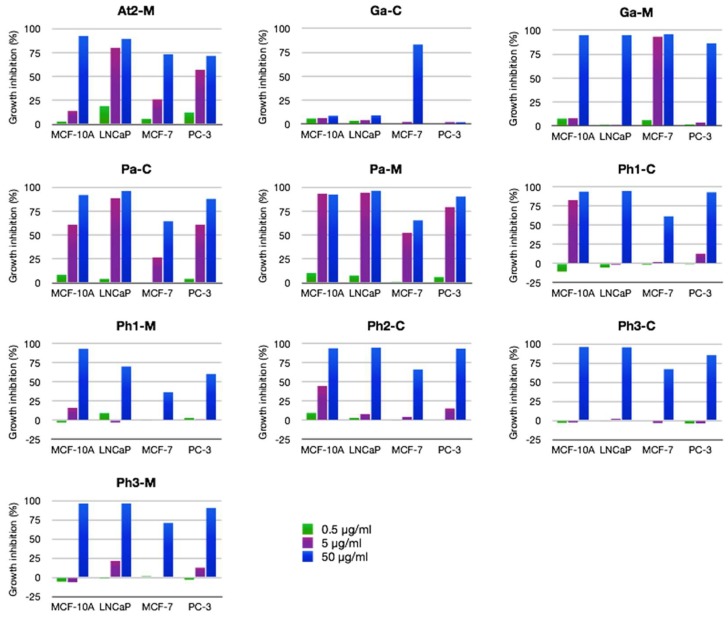
Dose-response results for anti-proliferative activity for selected specimen against tumorigenic (MCF-7) and non-tumorigenic (MCF-10A) breast cell lines and two tumorigenic prostate cell lines (LNCaP, PC-3) at 0.5 µg/mL (green), 5 µg/mL (purple), and 50 µg/mL (blue) concentration.

This screening led to the identification of strains producing antiproliferative effects on cell lines MCF-10A (breast cells), MCF-7 (breast cancer cells), LNCaP (prostate cancer cells), and PC-3 (prostate cancer cells). Dinoflagellates represent 73% of the samples evaluated. Overall, 15% of all dinoflagellates yielded extracts showing high antiproliferative activity in the initial screening at 50 µg/mL concentration in any cell line (Figure 3). Interestingly, the extract of cell free culture medium of *Gambierdiscus australes* (Ga-M) displayed activity >80% in all cell lines at the initial concentration while the biomass extract showed selectivity in the MCF-7 cell line. Similarly, *Alexandrium tamarense WE* medium (At2-M) showed high values of inhibition at 50 µg/mL. It is known that the water-soluble maitotoxins (MTXs) are among the most potent toxins and are produced by dinoflagellates of the genera *Gambierdiscus* and *Fukuyoa* [21]. This fact could explain the toxicity in the Ga-M extract. However, the extract Ga-C displayed significant selective activity on MCF-7 cells, which suggests that this extract might be interesting for further studies regarding its potential anticancer effects. On the other hand, the toxigenic activity of the genus *Alexandrium* is mainly due to neurotoxins such as saxitoxins, which are responsible of causing paralytic shellfish poisoning (PSP). However, the non-neurotoxic dinoflagellates of this genus can suppress copepod population growth [22]. These findings indicate the importance of extending the knowledge into the toxin profile of different species of *Alexandrium* searching for new antiproliferative compounds. Lastly, as discussed in the previous section, the *Prorocentrum* strains analyzed showed generalized toxicity. The samples related to the *Prorocentrum arenarium* strain (Pa-C and Pa-M) is the most active either at concentrations of 50 µg/mL or 5 µg/mL (Figure 4). 

Additionally, some extracts such as *Prymnesium faveolatum* medium (Pf-M) and *Emiliania huxleyi* cell (Eh-C) extracts enhance the growth of the cell lines particularly in the non-tumorigenic cell line MCF-10A. *Emiliania huxleyii* is a haptophyte that causes blooms and plays an important role in the global carbon cycle while the scarcely studied species of *Prymnesium* are responsible for massive fish deaths worldwide [23,24]. Although it is not the aim of this assay, the detection of extracts, which enhances cell growth could be useful for a preliminary test of significance for other types of bioactivities like anti-osteoporotic activity.

#### 2.2.3. Apoptotic Activity

**Figure 5:**
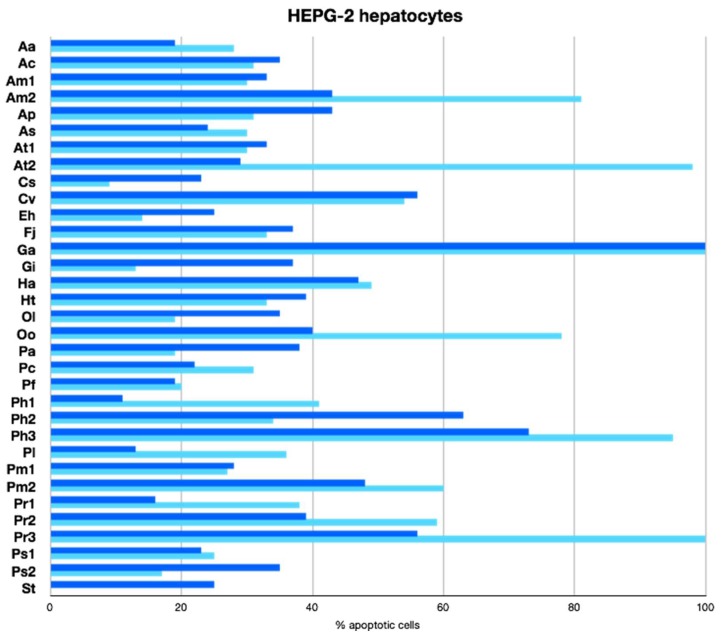
Apoptosis inducing activities (%) of HEPG-2 hepatocytes. Dark bars represent biomass extracts and light bar cell free culture medium extracts. Test carried out at 100 µg/mL.

To evaluate the potential of the extracts to induce apoptosis in an in vitro human liver cancer model, we conducted a primary screening of all of these extracts on the human hepatocellular carcinoma cell line HepG2 (ATCC^®^ HB-8065™). All the marine extracts were tested at 100 µg/mL (Figure 5). Of all the samples tested, 54% showed weak (20–49%) apoptosis-inducing activity and 9% showed moderated (50–79%) apoptosis-inducing activity. Surprisingly, 9% of the extracts showed further increased activity with 80–100% of apoptotic HepG2 cells at the concentration tested. The highest apoptotic potential was of the extracts of *Alexandrium minutum* (Am2-M), *Alexandrium tamarense WE* (At2-M), *Gambierdiscus australes* (Ga-C, Ga-M), *Prorocentrum hoffmannianum* 1031 (Ph3-M), and *Prorocentrum reticulatum* (Pr3-M).

Weak cell death of human lung carcinoma A549 and of colorectal carcinoma HT29 cell lines induced by *Alexandrium andersoni* raw cell extracts have been reported [25]. In addition, several other studies have demonstrated that some *Alexandrium* species caused detrimental or lethal effects on various marine living organisms inducing apoptotic responses [26,27]. Similarly, the toxic effects of the ciguatoxin P-CTX-1 on embryos of *Oryzias melastigma* have been investigated. P-CTX-1 may trigger the production of the p53 gene, which is the critical gene involved in the p53 pathway to promote apoptosis [28]. The primary screen results obtained for apoptotic-inducing activities of extracts of this genus suggested that these extracts might be interesting for further studies regarding their apoptotic potential. Lastly, okadaic acid (OA) and its derivatives produced by the *Prorocentrum* genus possess potent protein phosphatases (PPs) inhibitor activity. Studies with OA have suggested that several protein serine/threonine kinases and phosphatases are involved in the signaling pathways, which leads to apoptosis. OA derivatives with low or no inhibitory effect on PPs do not induce apoptosis, which suggests that the effect is specifically mediated by PPs inhibition such as PP2A [29].

## 3. Materials and Methods 

### 3.1. Microalgae Strains and Culture

Cultured strains were donated from the Spanish Institute of Oceanography (Vigo, Spain) by courtesy of Dr. Santiago Fraga. Stock cultures of strains (20 mL, 300 to 600 cells/mL) were gradually scaled up to 50 mL, 250 mL, and 5 L flasks containing 20 mL, 150 mL, and 3 L of seawater enriched with modified Guillard K medium, respectively. Composition of the modified K medium was the following: NaNO_3_, 882 µM, NH_4_Cl, 50 µM, NaH_2_PO_4_, 10 µM, TRIS, 1 mM, Na_2_EDTA.2H_2_O, 90 µM; FeEDTA.3H_2_O, 14.6 µM, MnCl_2_.4H_2_O, 0.9 µM, ZnSO_4_.7H_2_O, 0.08 µM, CoCl_2_.6H_2_O, 0.05 µM, Na_2_MoO_4_.2H_2_O, 0.03 µM, H_2_SeO_3_, 0.01 µM, thiamine-HCl, 0.3 µM, biotin, 2.1 nM, and B_12_, 0.37 nM (Chemicals required to make marine media were available from Sigma, St. Louis, MO, USA). All the cultures were incubated at 22 ± 2 °C with a light intensity of 60 μE/s m^2^ and with a light:darkness cycle of 18:6 hours. From all the scale up stages, inocula of an exponential growing phase were used to start the following cultures. Once the cultures grew in 5 L flasks up to a final volume of 15 L in an exponential growing phase, they were kept for 60 days under stress due to nutrient depletion. Then, cells were harvested by centrifugation (6000 g) and/or by filtration (GFF 0.22 µm).

### 3.2. Sample Preparation

Biomass and cell free culture medium were processed separately as follows:

Biomass was extracted with methanol under sonication (5 × 500 mL). Afterwards, the methanol extract was filtered and solvent removed in vacuo. Crude extracts were transferred to vials for biological evaluation.

Cell free culture media was passed through a Diaion HP20 (6 Ø × 40 cm) column using a flow rate of 1 bed-volume/h. After that, the column was washed with 500 mL of distilled water and the organic compounds were desorbed with methanol (4 × 1.5 L). The methanol extract was filtered and the solvent was removed in vacuo. 

### 3.3. Antibacterial and Antifungal Assays

Antimicrobial properties were evaluated against bacterial strains *Staphylococcus aureus* (Gram-positive, ATCC 25923), *Enterococcus faecalis* (Gram-positive, ATCC 29212), *Escherichia coli* (Gram-negative, ATCC 25922), and a fungal strain *Candida albicans* (ATCC 90028) obtained from Microbiologics Inc. (St. Cloud, USA). Bacterial stock cultures were maintained on Mueller-Hinton agar (Beckton Dickinson, Franklin Lakes, NJ, USA) and fungal culture on Saboraud Dextrose agar (SDA). Before the assay, bacterial suspensions were inoculated into Mueller-Hinton broth (MHB) and incubated at 37 °C for 16 to 20 h at 100 rpm. *Candida* suspension for the assay was prepared by suspending colonies from a fresh SDA culture into sterile 0.9% NaCl. Antimicrobial activity was tested using a broth micro dilution method in a 96-well format according to EUCAST and CLSI guidelines. Final inoculum of 5 × 10^5^ colony-forming units (CFU)/mL in MHB was used for the bacteria and 2.5 × 10^3^ CFU/mL in RPMI-1640 media (with l-glutamine, *w*/*o* NaHCO_3_ and supplemented with 2% glucose and 0.165 M MOPS, buffered to pH 7, Lonza, CH) for *Candida*. DMSO stocks of extracts were first diluted into the assay media and then mixed with the bacteria or fungi suspension on the assay plate. Plates were incubated at 37 °C for 24 h (bacteria) or at 28 °C for 48 h (fungi) at 500 rpm. Absorbance values measured at 620 nm were used for calculating the percentage of inhibition by comparing it to untreated controls. Ciprofloxacin was used as a positive control in antibacterial assays (MIC_90_ values for *S. aureus*, *E. coli*, and *E. faecalis* were 0.5, 0.016, and 1 µg/mL, respectively). Amphotericin B was used as a positive control in antifungal assays (MIC_90_: 0.5 µg/mL). Primary testing of extracts was carried out at a final concentration of 100 µg/mL (*n* = 3). 

### 3.4. Antiviral Assays

A replicon model of the Chikungunya virus (CHIKV) was primarily used for assessing antiviral properties of the extracts. The assay is based on a stable BHK21 cell line (BHK-CHIKV-NCT) previously described by Pohjala et al. [30]. The BHK-CHIKV-NCT cells were maintained at 37 °C, 5% CO_2_, and 95% humidity in Dulbecco’s Modified Eagle’s Medium (DMEM) with high glucose and l-glutamine (Gibco®, Paisley, UK) supplemented with 7.5% fetal bovine serum (FBS), 2% tryptose-broth phosphate, 1 mM sodium pyruvate, 100 IU/mL penicillin, 100 µg/mL streptomycin, and 5 µg/mL puromycin. For the assay, cells were seeded onto 96-well plates, 40,000 cells/well, and incubated for 24 h at 37 °C. DMSO stocks of extracts were diluted into an assay medium consisting of DMEM with high glucose and l-glutamine (Gibco®, Paisley, UK) supplemented with 5% fetal bovine serum (FBS), 1 mM sodium pyruvate, 100 IU/mL penicillin, and 100 µg/mL streptomycin and added to the cells. After 48 h exposure, the *Rluc* expression was determined by using a *Renilla* luciferase assay kit (Promega, Madison, WI, USA), according to the manufacturer’s instructions. Luminescence was measured using a Varioskan Flash plate reader (Thermo Fisher Scientific, Vantaa, Finland). The percentage of inhibition of the viral replicon was calculated by comparing the sample signal to the yielded maximum signal (DMSO vehicle in an assay medium). 6-Azauridine was used as a positive control (IC_50_ = 2 µM). Primary testing of extracts was carried out at a final concentration of 100 µg/mL (*n* = 3). To rule out false positives due to host cell cytotoxicity, cell viability after sample exposure was evaluated by ATP quantitation with CellTiter GLO^®^ (Promega, Madison, WI, USA), according to the manufacturer’s instructions. Sample exposure was carried out by mimicking the conditions of the replicon assay.

### 3.5. Antiproliferative Activity 

Cell culture: LNCaP cells were purchased from the Deutsche Sammlung von Microorganismen und Zellkulturen GmbH. PC-3 and MCF-10A were from ATCC (Manassas, VA, USA). MCF-7 cells were obtained from the Interlab Cell Line Collection (ICLC, Genova, Italy). MCF-7 cells were cultured in Dulbecco’s modified Eagle’s medium (1 g/L glucose) and supplemented with 10% fetal bovine serum, 2 mM l-glutamine, and 1% penicillin/streptomycin. LNCaP and PC-3 cells were grown in RPMI-1640, 10% serum, 1% penicillin/streptomycin, and 2 mM l-glutamine. MCF-10A were grown in 1:1 DMEM (4.5 g Glucose/L) and HAM F-12, 5% horse serum, 5 µg/mL hydrocortisone, 20 ng/mL EGF, 100 ng/mL choleratoxin, 2 mM l-glutamine, and 10 µg/mL insulin. All cells were grown in 37 °C/5% CO_2_.

High throughput anticancer assays: Samples that were not provided in readymade solutions were weighed and dissolved to DMSO in 50 or 20 mg/mL stock concentrations depending on the amount provided. Samples were transferred into 384 well master plates and subsequently processed with a pipetting robot for 1:10 serial dilutions (20 mg/mL, 2 mg/mL, and 0.2 mg/mL) for high-throughput screening. A total of 100 nL of the compound solutions were transferred into white 384-well assay plates (Greiner, Kremsmünster, Austria) in four replicates for readymade assay plates to be stored in the freezer until used for the screens (final assay concentrations 50, 5, and 0.5 µg/mL). A CellTiter GLO^®^ (CTG, Promega, Madison, WI, USA) proliferation assay for two prostate cancer cell lines (LNCaP and PC-3) and one breast cancer cell line (MCF-7) was carried out in order to assess the anticancer effects of the extracts/compounds. For 72 out of the 100 samples, a proliferation screen was also carried out in non-tumorigenic epithelial MCF-10A cells to better interpret the specific anticancer activity. DMSO and water were used as a negative and staurosporine and daunorubicine (5 µg/mL, 0.5 µg/mL, and 0.05 µg/mL of both) were used as positive controls. Cells (1500 MCF-10A, 1700 PC-3, 2000 MCF-7, and LNCaP cells/well) were plated in 40 µL of the culture medium into the assay plates on top of the compounds. After 72 h of incubation in 37 °C/5% CO_2_, 20 µL of a CTG reagent was added, plates were placed in an orbital shaker for 30 min for lysis, and the luminescence was measured with the Envision Multilabel reader (PerkinElmer, Waltham, MA, USA). The screens were carried out in two biological replicates in two separate screen sets. The average signals for the four replicate samples in duplicate screens were calculated and compared to the averages of the control samples (DMSO or water). The samples that qualified as anti-proliferative hits inhibited cell viability by at least three SDs from the average of the controls. The screens were technically successful. DMSO controls showed less than 10% CV. The positive controls were very effective and the replicate samples showed very low variation. Due to the fact that the replicate biological screens correlated so well in early screens, only one screen was carried out in the later experiments.

### 3.6. Apoptotic Activity

Cell based experiments: The human hepatocellular carcinoma cell line HepG2 (HB-8065) was obtained from the American Type Culture Collection (Maryland, MA, USA) and maintained in DMEM and supplemented with 10% FBS, P/S (100 unit/mL and 100 µg/mL), and glutamine (2 mM). HepG2 cells were grown in a humidified atmosphere with 5% CO_2_ at 37 °C in 75 cm^2^ flasks up to 70% to 80% confluence prior to treatment. Marine extracts were diluted in DMSO. For our purpose, cell lines were treated with the appropriate working concentrations (100 µg/mL) and mixed with the cell culture medium. The highest concentration of DMSO (for treated and untreated cells) never exceeded 0.25% (*v*/*v*) to avoid side effects like cell toxicity or induction of differentiation.

Apoptosis rate analysis by an annexin V binding assay: Apoptosis activities were assessed by evaluating the externalization of phosphatidylserine and nucleus labelling by propidium iodide. For that purpose, cells were cultured, treated, or not with our extracts in 96-well plates. Cell were washed with PBS, trypsinized, centrifuged, and re-suspended in the preserved supernatant of the first wash in order to keep all non-adherent apoptotic cells present. A minimum of 2000 cells was acquired per sample and analyzed on the InCyte software (Guava, Millipore Merck, Burlington, MA, USA). To discriminate between negative and positive events in the analysis, a non-stained control sample from each culture condition always accompanied acquisition of the stained cells to define the cut off. A negative control i.e., sample with cells without compounds but with the same percentage of DMSO (*v*/*v*) as for diluted compounds was included in each experiment. Celastrol was used as a positive control for apoptotic assays. Apoptosis rates were assessed by capillary cytometry (Guava EasyCyte Plus, Millipore Merck, Burlington, MA, USA) using Annexin V-FITC (ImmunoTools, Friesoythe, Germany) and propidium iodide (MiltenyiBiotec Inc., Auburn, CA USA), according to the manufacturer’s recommendations. Gates were drawn around the appropriate cell populations using a forward scatter (FSC) versus a side scatter (SSC) acquisition dot plot to exclude debris. Cytometers performances are checked weekly using the Guava Easy Check Kit 4500-0025 (Merck, Millipore, Burlington, MA, USA).

## 4. Conclusions

In summary, a bioprospecting study on 33 microalgae strains was carried out. A total of 65 extracts obtained from biomass and cell free culture medium were analyzed in several selected assays to provide information on antimicrobial, anti-proliferative, and apoptotic activities through an interlaboratory collaboration. The wide range of marine micro-algal species were tested for the first time. It complements the few studies performed on it until now [31,32,33,34,35,36].

**Figure 6:**
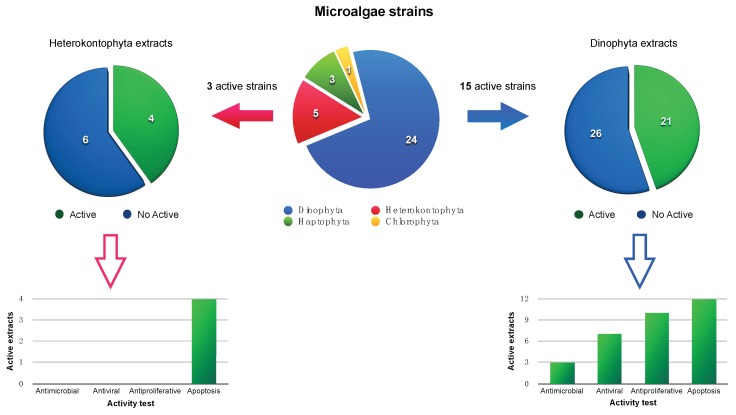
Distribution of the microalgae strains evaluated in this study as a function of their phylum including a proportion of active or inactive extracts in each case.

In all cases, when the samples showed activity larger than 50% above the control levels were estimated positive. The comprehensive results are represented in Figure 6. Altogether, 21 extracts of phylum Dinophyta (corresponding to 15 strains) and four extracts of phylum Heterokontophyta (three strains) were identified to be active against the panel of assays even though the samples belonging to Heterokontophyta only showed moderate apoptotic activity. Furthermore, eight in 24 dinoflagellates strains displayed significant activity (>80%). Among them, *Prorocentrum hoffmannianum* 1029 and 1030 exhibit high values of anti-proliferative effects against cancer cell lines at 50 µg/mL while *Prorocentrum arenarium* was the most active either at 50 µg/mL or 5 µg/mL. *Prorocentrum reticulatum (Pr-3*) induces 100% apoptosis in HepG2 cells as well as *Alexandrium tamarensis WE* and *Gambierdiscus australes*, which showed both promising anti-proliferative and apoptotic responses. Therefore, these cultured dinoflagellates would be excellent objectives for a deeper chemical and pharmacological study. In addition, extracts from *Prorocentrum hoffmannianum 1031* displayed a general potent cytotoxicity against all assays in the range of 90% to 100% and would be interesting to study further to know what causes the effects.

The outcome of this study provides additional scientific data for the selection of several microalgae strains for further investigation and offers support for the continued research for the development of new potential drugs for human therapeutics from cultured microalgae.

## Supplementary Materials

Supplementary materials are available online at http://www.mdpi.com/1660-3397/16/9/317/s1. Table S1: Strain codes and isolation zone for bioprospecting microalgae, Table S2: Anti-microbial, antiviral, anti-proliferative and apoptotic results for 65 extracts of marine microalgae. Results are expressed as a percentage effect compared to the untreated control. Extracts were tested at a final concentration of 100 g/mL (antimicrobial, antiviral, and apoptotic) and 50 g/mL (anti-proliferative). For clarity, color scale has been applied to highlight the differences in values.
