# Supplementary material for: Marine Microalgae: Promising Source for New Bioactive Compounds
Source: Mar Drugs. 2018 Sep 6;16(9):317. doi: 10.3390/md16090317 (PMC6164378; doi:10.3390/md16090317)
Supplement: Supplementary file 1 [file marinedrugs-16-00317-s001.pdf]

Appendix A

Table S1. Strain codes and isolation zone for bioprospecting microalgae.

Strain ID	Strain	Isolation Zone	[1]Sample Code
		**Phylum Dinophyta**	
VGO626	*Akashiwo sanguinea*	Kavala Harbour, Greece	As-C/As-M
PA8V	*Alexandrium affine*	La Línea de la Concepción, Spain	Aa-C / Aa-M
VGO566	*Alexandrium catenella TA*	Puerto de Tarragona, Spain	Ac-C / Ac-M
VGO661	*Alexandrium margalefi*	Alfacs, Delta del Ebro, Spain	Am1-C / Am1-M
AL1V	*Alexandrium minutum*	Ría de Vigo, Spain	Am2-C / Am2-M
VGO956	*Alexandrium peruvianum*	Palamós, Gerona, Spain	Ap-C / Ap-M
CNRATAA1	*Alexandrium tamarense ME*	Mar Piccolo di Tarento, Puglia, Italy	At1-C / At1-M
PE1V	*Alexandrium tamarense WE*	Ría de Vigo, Spain	At2-C / At2-M
VGO1046	*Gambierdiscus australes*	Honolulu, Hawaii, USA	Ga-C / Ga-M
GY1VA	*Gymnodinium impudicum*	Puerto de Valencia, Spain	Gi-C / Gi-M
VGO1055	*Heterocapsa triquetra*	N.D.	Ht-C / Ht-M
VGO614	*Ostreopsis ovata*	Bahía de Abra, Madeira, Portugal	Oo-C / Oo-M
VGO776	*Prorocentrum arenarium*	Punta del Hidalgo, Tenerife, Spain	Pa-C / Pa-M
VGO834	*Prorocentrum cassubicum*	Almerimar, Almería, Spain	Pc-C / Pc-M
VGO1029	*Prorocentrum hoffmannianum 1029*	La Puntilla, Gran Canaria, Spain	Ph1-C / Ph1-M
VGO1030	*Prorocentrum hoffmannianum 1030*	La Puntilla, Gran Canaria, Spain	Ph2-C / Ph2-M
VGO1031	*Prorocentrum hoffmannianum 1031*	La Puntilla, Gran Canaria, Spain	Ph3-C / Ph3-M
VGO777	*Prorocentrum levis*	Punta del Hidalgo, Tenerife, Spain	Pl-C / Pl-M
PM1V	*Prorocentrum micans*	Ría de Vigo, Spain	Pm1-C / Pm1-M
AND1V	*Prorocentrum mínimum*	Río San Pedro, Cádiz, Spain	Pm2-C / Pm2-M
PR1V	*Prorocentrum rostratum*	Ría de Vigo, Spain	Pr1-C / Pr1-M
VGO404	*Protoceratium reticulatum*	Salton Sea, California, USA	Pr2-C/ Pr2-M
CCMP1720	*Protoceratium reticulatum*	Biscayne Bay, Miami, Florida, USA	Pr3-C / Pr3-M
S3V	*Scrippsiella trochoidea*	Ría de Vigo, Spain	St-C / --
		**Phylum Heterokontophyta**	
VGO1039	*Chattonella subsalsa*	Bigelow CCMP Collection	Cs-C / Cs-M
VGO1038	*Chatonella verruculosa*	N.D.	Cv-C / Cv-M
VGO1043	*Fibrocapsa japonica*	Bigelow CCMP Collection	Fj-C / Fj-M
HA1V	*Heterosigma akashiwo*	Ría de Arousa, Spain	Ha-C / Ha-M
VGO1036	*Olisthodiscus luteus*	SCCAP Collection	Ol-C / Ol-M
		**Phylum Haptophyta**	
EH02V	*Emiliania huxleyi*	Cabo Estai, Vigo, Spain	Pf-C / Pf-M
VGO1040	*Prymnesium*	N.D.	Ps1-C / Ps1-M
VGO557	*Prymnesium faveolatum*	Pixavaques, Gerona, Spain	Eh-C / Eh-M
		**Phylum Chlorophyta**	
PY01V	*Pyramimonas sp*	Lorbé, A Coruña, Spain	Ps2-C / Ps2-M

[1]Extract from biomass: species code-C. Extract from cell free culture medium: species code-M.
N.D.: No determined. CCMP: Center for Cultures of Marine Phytoplankton. SCCAP: Scandinavian Culture Collection for Algae and Protozoa.

Table S2. Anti-microbial, antiviral, anti-proliferative and apoptotic results for 65 extracts of marine microalgae. Results are expressed as a percentage effect compared to the untreated control. Extracts were tested at a final concentration of 100 µg/mL (antimicrobial, antiviral, and apoptotic) and 50 µg/mL (anti-proliferative). For clarity, color scale has been applied to highlight the differences in values.

	Antimicrobial				Antiviral	Anti-Proliferative				Apoptotic
**Extract Code**	***Enterococcus faecalis*** **(ATCC 29212)**	***Escherichia coli*** **(ATCC 25922)**	***Staphylococcus aureus*** **(ATCC 25923)**	***Candida albicans*** **(ATCC 90028)**	**CHIKV replicon model**	**MCF-10A (breast cells)**	**LNCaP (prostate cancer cells)**	**MCF-7 (breast cancer cells)**	**PC-3 (prostate cancer cells)**	**HEPG-2 hepatocytes (% apopt cells)**
As-C	0	6	−8	−15	60	2	9	4	0	24
As-M	2	3	−29	−5	0	7	8	4	1	30
Aa-C	−24	3	16	−3	51	5	16	5	9	19
Aa-M	−29	−2	−31	5	21	3	11	2	2	28
Ac-C	16	−19	−65	−77	8	6	13	0	3	35
Ac-M	4	7	−47	7	23	3	12	1	6	31
Am1-C	20	4	−24	−23	28	2	16	7	11	33
Am1-M	11	−2	−20	−11	68	4	14	11	18	30
Am2-C	10	−12	−207	−5	29	−2	−2	7	−2	43
Am2-M	8	6	−52	−11	52	3	3	4	4	81
Ap-C	26	−14	−28	−3	0	4	13	1	5	43
Ap-M	10	2	−48	23	4	3	8	0	2	31
At1-C	3	6	−108	−71	8	1	4	7	0	33
At1-M	0	6	−137	6	25	2	4	6	2	30
At2-C	−18	1	20	2	56	5	15	5	12	29
At2-M	−24	2	−34	−19	82	96	89	73	72	98
Ga-C	17	−20	30	5	NT	9	9	83	2	100
Ga-M	8	−14	8	23	NT	94	95	96	86	100
Gi-C	−14	4	−18	−17	0	2	13	3	4	37
Gi-M	−6	1	−35	−29	0	3	10	−1	1	13
Ht-C	3	7	−33	−23	0	2	14	0	4	39
Ht-M	−15	2	−30	−30	0	1	10	2	3	33

−200	−100	0	50	100

NT: No tested.

Table S2. Continuation.

	Antimicrobial				Antiviral	Anti-Proliferative				Apoptotic
**Extract Code**	***Enterococcus faecalis* (ATCC 29212)**	***Escherichia coli* (ATCC 25922)**	***Staphylococcus aureus* (ATCC 25923)**	***Candida albicans* (ATCC 90028)**	**CHIKV replicon model**	**MCF-10A (breast cells)**	**LNCaP (prostate cancer cells)**	**MCF-7 (breast cancer cells)**	**PC-3 (prostate cancer cells)**	**HEPG-2 hepatocytes (% apopt cells)**
Oo-C	−4	2	−117	−8	NT	5	9	2	3	40
Oo-M	−8	7	−126	−32	9	−1	1	7	−2	78
Pa-C	21	−11	3	60	NT	92	96	64	88	38
Pa-M	0	−3	11	6	NT	93	96	65	90	19
Pc-C	9	2	6	−36	0	−8	0	8	−3	22
Pc-M	−13	4	−14	50	NT	5	8	1	1	31
Ph1-C	3	3	7	−17	0	94	95	62	93	11
Ph1-M	7	9	25	−10	8	94	71	37	61	41
Ph2-C	12	−9	2	22	NT	94	95	67	94	63
Ph2-M	−4	5	−21	9	5	0	5	11	2	34
Ph3-C	15	−3	−179	14	89	96	96	67	85	73
Ph3-M	100	21	55	98	96	96	97	71	91	95
Pl-C	−5	−4	−3	−19	NT	3	8	3	2	13
Pl-M	−119	−8	2	−23	NT	4	29	3	30	36
Pm1-C	−2	3	10	−24	0	−8	−3	−2	−3	28
Pm1-M	3	8	8	4	0	−11	−6	−11	−5	27
Pm2-C	−8	4	−142	−14	27	−1	2	7	0	48
Pm2-M	−14	8	−65	0	21	−2	1	6	−2	60
Pr1-C	0	13	6	26	0	−11	3	5	0	16
Pr1-M	−8	8	12	−38	0	−11	2	1	−1	38
Pr2-C	0	6	1	6	NT	5	8	1	1	39
Pr2-M	−3	−11	37	−34	NT	8	10	1	2	56

−200	−100	0	50	100

NT. Not tested.

Table S2. Continuation.

Extract Code	Antimicrobial				Antiviral	Anti-Proliferative				Apoptotic
	Enterococcus faecalis (ATCC 29212)	*Escherichia coli* (ATCC 25922)	*Staphylococcus aureus* (ATCC 25923)	*Candida albicans* (ATCC 90028)	CHIKV replicon model	MCF-10A (breast cells)	LNCaP (prostate cancer cells)	MCF-7 (breast cancer cells)	PC-3 (prostate cancer cells)	HepG-2 hepatocytes (% apopt cells)
Pr3-C	−15	5	7	−16	NT	4	6	3	4	59
Pr3-M	−23	−8	−2	−12	NT	3	25	7	15	100
St-C	1	1	11	−17	0	−9	−3	−4	−4	25
Cs-C	2	−1	−2	−18	0	−1	11	2	−2	23
Cs-M	1	−1	−41	−18	7	0	4	2	1	9
Cv-C	4	−11	−133	−35	0	−1	−2	11	2	56
Cv-M	−9	6	−110	0	0	−1	2	9	−1	54
Fj-C	4	10	−141	−1	16	−2	0	12	0	37
Fj-M	−5	7	−105	5	9	−4	0	7	−2	33
Ha-C	3	−8	−186	1	1	0	1	11	0	47
Ha-M	5	7	−95	21	14	−3	1	8	−1	49
Ol-C	0	6	−50	−27	48	2	14	3	1	35
Ol-M	6	4	−41	−39	8	6	9	1	0	19
Pf-C	4	4	10	−11	0	−16	−1	8	0	19
Pf-M	4	6	6	−6	0	−15	−6	−2	−3	20
Ps1-C	−7	4	13	−50	0	−12	−4	1	−4	23
Ps1-M	−1	5	12	12	0	−15	−9	−4	−6	25
Eh-C	3	7	7	−15	0	−10	−2	−5	−3	25
Eh-M	8	6	11	−14	0	−9	0	−9	−3	14
Ps2-C	−107	6	2	−24	NT	6	9	1	1	35
Ps2-M	13	3	3	−75	5	−11	−4	2	−2	17

−200	−100	0	50	100

NT: Not tested.
